# METABOLITES ASSOCIATED WITH HIGH VERSUS LOW WALKING ABILITY AMONG COMMUNITY-DWELLING OLDER MEN AND WOMEN

**DOI:** 10.1093/geroni/igz038.2386

**Published:** 2019-11-08

**Authors:** Megan M Marron, Stacy G Wendell, George C Tseng, Robert M Boudreau, Adam J Santanasto, Clary Clish, Joseph M Zmuda, Anne B Newman

**Affiliations:** 1 University of Pittsburgh, Pittsburgh, Pennsylvania, United States; 2 Broad Institute, Cambridge, Massachusetts, United States; 3 Department of Epidemiology, University of Pittsburgh; Pittsburgh, Pennsylvania, United States

## Abstract

Low walking ability is highly prevalent with advanced age and associated with a higher risk of major adverse health outcomes. Metabolomics may help better characterize differences among older adults with vastly different walking abilities and provide insight into altered metabolic processes underlying age-related declines in physical functioning. Here, we sought to identify metabolites associated with high versus low walking ability using a nested case-control study of 120 community-dwelling adults ages 79-95 (40% men, 10% black) from the Cardiovascular Health Study (CHS) All Stars study. Participants with high versus low walking ability were matched one-to-one on age, gender, race, and fasting time. Using liquid chromatography-mass spectrometry, 569 metabolites were identified in overnight-fasting plasma. High versus low walking ability was defined as the best versus worst tertile of gait speed (≥0.9 versus <0.7 meters/second) and Walking Ability Index scores (7-9 versus 0-1). Ninety-six metabolites were associated with walking ability extremes (p<0.05, false discovery rate<30%), where 24% were triacylglycerols. Triacylglycerols containing mostly polyunsaturated fatty acids (e.g., omega-3) were higher, whereas those containing mostly saturated/monounsaturated fatty acids were lower among those with high versus low walking ability. Arginine and proline metabolism was a top pathway identified. Body mass index partly explained the association between a subset of metabolites and walking ability extremes. These findings may partly reflect pathways implicating modifiable risk factors including excess dietary lipids and lack of physical activity, which contribute to obesity and cause further alterations in metabolic pathways, potentially leading to age-related declines in walking ability in this cohort.

